# Assembly and Characterization of HBc Derived Virus-like Particles with Magnetic Core

**DOI:** 10.3390/nano9020155

**Published:** 2019-01-26

**Authors:** Jakub Dalibor Rybka, Adam Aron Mieloch, Alicja Plis, Marcin Pyrski, Tomasz Pniewski, Michael Giersig

**Affiliations:** 1 Center for Advanced Technology, Adam Mickiewicz University in Poznań, Umultowska 89C, 61-614 Poznań, Poland; amieloch@amu.edu.pl (A.A.M.); alkaplis@o2.pl (A.P.); giersig@amu.edu.pl (M.G.); 2 Faculty of Chemistry, Adam Mickiewicz University in Poznań, Umultowska 89B, 61-614 Poznań, Poland; 3 Institute of Plant Genetics, Polish Academy of Sciences, Strzeszyńska 34, 60-479 Poznań, Poland; mpyr@igr.poznan.pl (M.P.); tpni@igr.poznan.pl (T.P.); 4 Institute of Experimental Physics, Freie Universität Berlin, Arnimallee 14, 14195 Berlin, Germany

**Keywords:** virus-like particles, VLPs, hepatitis B virus capsid protein, HBc, viral self-assembly, magnetic core, HBcAg

## Abstract

Core-virus like particles (VLPs) assembly is a kinetically complex cascade of interactions between viral proteins, nanoparticle’s surface and an ionic environment. Despite many in silico simulations regarding this process, there is still a lack of experimental data. The main goal of this study was to investigate the capsid protein of hepatitis B virus (HBc) assembly into virus-like particles with superparamagnetic iron oxide nanoparticles (SPIONs) as a magnetic core in relation to their characteristics. The native form of HBc was obtained via agroinfection of *Nicotiana benthamiana* with pEAQ-HBc plasmid. SPIONs of diameter of 15 nm were synthesized and functionalized with two ligands, providing variety in ζ-potential and hydrodynamic diameter. The antigenic potential of the assembled core-VLPs was assessed with enzyme-linked immunosorbent assay (ELISA). Morphology of SPIONs and core-VLPs was evaluated via transmission electron microscopy (TEM). The most successful core-VLPs assembly was obtained for SPIONs functionalized with dihexadecyl phosphate (DHP) at SPIONs/HBc ratio of 0.2/0.05 mg/mL. ELISA results indicate significant decrease of antigenicity concomitant with core-VLPs assembly. In summary, this study provides an experimental assessment of the crucial parameters guiding SPION-HBc VLPs assembly and evaluates the antigenicity of the obtained structures.

## 1. Introduction

Virus-like particles (VLPs) are non-infectious and non-replicating supramolecular assemblies composed of single or multiple viral proteins, which closely resemble native virions [1]. VLPs display a unique set of immunological characteristics that render them highly potent for vaccine development such as: nanometer range size, multivalent and highly repetitive surface geometry, the ability to elicit both innate and adaptive immune response [2]. Due to favorable surface morphology and a wide range of possible modifications, VLPs have been successfully used as a platform for multivalent vaccine creation [3,4,5]. Several VLP-based vaccines are currently commercially available (e.g., Cervarix®, Gardasil®, Sci-B-Vac™, Mosquirix™) with more undergoing clinical trials [6]. VLPs’ applicability is not limited to their immunogenic properties. Some of the use cases include: highly selective and sensitive nanobiosensor for troponin I detection, light-harvesting VLPs for use in photovoltavic or photocatalytic devices, nanofiber-like VLPs for tissue regenerating materials, nanocontainers and nanoreactors [7,8,9,10,11].

Hepatitis B virus (HBV) is an enveloped, icosahedral, cDNA virus that belongs to the Hepadnaviridae family. The virion has a diameter of 42 nm and is composed of a lipid envelope with hepatitis B virus surface antigen (HBsAg) and inner nucleocapsid consisting of hepatitis B virus capsid protein—HBc (named also HB core antigen, HBcAg) [12]. HBc consists of 183–185 amino acids of which 149 N-terminal amino acids form an assembly domain and 34 amino acids form C-terminal arginine-rich domain (CTAD) required for the packaging of nucleic acid [13]. HBc is a homodimeric protein that has the ability to self-assemble into icosahedral and fenestrated T = 4 (120 dimers) and T = 3 (90 dimers) capsids with respective outer diameter of 34 and 30 nm [14]. T = 4 capsid is a dominant product of a wild type HBc in vitro self-assembly (~95%) [15]. HBc capsid is highly immunogenic and has been shown to induce both B- and T-cell response [16].

Superparamagnetic iron oxide nanoparticles (SPIONs) exhibit properties, such as high magnetic susceptibility, high saturation magnetization and low toxicity [17,18,19]. Due to the aforementioned properties, high-yield synthesis methods and a wide array of available surface modifications, SPIONs can be utilized in: magnetic bioseparation, magnetic hyperthermia, targeted drug delivery, in diagnostics as magnetic resonance imaging (MRI) contrast agents, etc. [20,21,22,23].

Introduction of SPIONs as the core of VLPs has been performed successfully with several viral proteins of different origin [24,25,26]. Magnetic core adds a multitude of advantageous properties. It allows for post-assembly magnetic bioseparation, which may be crucial for large scale production [27]. It also improves cellular uptake and magnetic relaxivities resulting in higher resolution MRI images, which combined with in vivo tracking may provide essential data regarding VLPs biodistribution [28]. Functionalized core can act as a substitute for native nucleic acid, and therefore, govern the process of protein recruitment and organization during self-assembly. Rational core design can be used to facilitate the assembly and enhance such parameters as, e.g., physicochemical stability, mechanical elasticity, capacity to withstand desiccation and long-term storage. On the other hand, HBc VLPs have been shown to be potent epitope carriers [3,29].

In the study by Shen et al., HBc was genetically engineered into a truncated version, deprived of 34 C-terminal amino acids responsible for nucleic acid packaging. The removed part was replaced by six consecutive histidine residues (His-tag). Fe_3_O_4_ nanoparticles functionalized with nickel-nitrilotriacetic acid (nickel-NTA) chelate were used as the core. The VLPs assembly was driven by the affinity of histidine tags to the nickel-NTA chelate [28]. This study prompted us to investigate whether native HBc VLPs assembly can be successful, without resorting to genetic engineering of HBc protein.

The main objective of this study was to investigate whether provided SPION surface modification is sufficient for SPION-HBc assembly. Even though HBc subunits exhibit an ability to assemble in the absence of genetic material, electrostatic interactions between positively charged CTAD of the capsid and negatively charged nucleic acid have a major influence on the assembly process [30]. Therefore, to mimic native electrostatic interactions, negatively charged ligands were chosen for SPIONs functionalization: dihexadecyl phosphate (DHP) and PL-PEG-COOH. Both compounds were successfully used in our previous study regarding the influence of ligand charge and length on the assembly of Brome mosaic virus derived virus-like particles with magnetic core [31].

## 2. Materials and Methods

### 2.1. Reagents

Oleic acid (technical grade 90%), Iron (III) acetylacetonate (97%), Sodium chloride (99%), Dihexadecyl phosphate (90%), 1-Octadecene (90%), 2-Butanol (95.5%), Trizma® hydrochloride (99%), Calcium chloride (97%), Magnesium sulfate (99.5%), Glycine (99%), Glycerol (99%), Urea (98%), 2-(N-Morpholino)ethanesulfonic acid (99%), Sucrose (99.5%), Sigma-Aldrich (Poznan, Poland). Toluene (99.5%), n-Hexane (99%), Chloroform (98.5%) and Hydrochloric acid (30–35%), Avantor (Gliwice, Poland). 1,2-Distearoyl-sn-glycero-3-phosphoethanolamine-N-[carboxy-(polyethyleneglycol)-2000] (ammonium salt) (PL-PEG-COOH, 2000 Da PEG (99%), Avanti, Alabaster, AL, USA). Snakeskin® Dialysis Tubing, 10K MWCO, 22 mm, Thermo Fisher Scientific (Waltham, MA, USA). All chemicals were used as received. Water was purified with Hydrolab HLP5 instrument (0.09 μS/cm, Straszyn, Poland).

### 2.2. Superparamagnetic Iron Oxide Nanoparticles (SPIONs) Synthesis

Spherical iron oxide nanoparticles were synthesized via thermal decomposition of iron (III) acetylacetonate Fe(acac)_3_ [32]. Briefly, 6 mmol of Fe(acac)_3_ and 18 mmol of oleic acid were dissolved in 40 mL of 1-octadecene. The reaction was performed with continuous stirring and nitrogen flow. Temperature of the solution was increased to 220 °C and maintained for 1 h. Subsequently, the temperature was increased further to 320 °C and maintained for 1 h. After synthesis, the solution was left to cool down to ambient temperature and 200 mL of washing solution (3:1 *v*/*v* of 2-butanol and toluene) was added. The obtained mixture was placed on a neodymium magnet and left overnight to allow nanoparticles to precipitate. Supernatant was discarded and replaced with fresh washing solution. Sonicating bath was used to resuspend nanoparticles. The washing step was performed thrice. In the final step, nanoparticles were suspended in 20 mL of chloroform. Concentration of the nanoparticles was estimated by dried sample weighing.

### 2.3. SPIONs Functionalization

PL-PEG-COOH functionalization was performed as per a method published elsewhere [24], with minor modifications. Briefly, 3.0 mg of PL-PEG-COOH were added to 5 mL of 1.0 mg/mL SPIONs chloroform solution. The sample was briefly sonicated in a sonic bath and left open for chloroform evaporation. The obtained waxy solid was heated for 1 min in an 80 °C water bath. The following step was adding 5 mL of miliQ water and vortexing the sample to enhance micelles formation. Subsequently, the sample was washed thrice with chloroform to remove unbound PL-PEG-COOH. Finally, water phase containing functionalized SPIONs was collected and filtered through 0.22 μm pores. Concentration of the SPION-PEG nanoparticles was measured via thermogravimetric analysis described below.

DHP functionalization was performed as per a method published elsewhere [31], with minor modifications. Briefly, 10.0 mg of dihexadecyl phosphate were added to 20 mL of hexane and dissolved with heat-assisted magnetic stirring (75 °C, ca. 10 min). After DHP dissolution, a chloroform solution containing 10.0 mg of synthesized iron oxide nanoparticles coated with oleic acid was added. The mixture was shortly sonicated and 80 mL of water were added. Subsequently, the obtained two phase solution was briefly vortexed and sonicated until the water phase became turbid. In the next step, the solution was placed in a sonicating bath for 3–4 h with no temperature control exercised. After functionalization, the solution was left overnight to allow for phase separation. The Bobtom phase was collected and placed near neodymium magnet for 24 h to separate functionalized nanoparticles from the solution. The obtained precipitate was collected, suspended in 2 mL of miliQ water and filtered through 0.22 μm pores. Concentration of the SPION-DHP nanoparticles was measured via thermogravimetric analysis described below.

### 2.4. Concentration Measurement via Thermogravimetric Analysis

Thermogravimetric analysis was performed to measure concentrations of the functionalized SPIONs. The analysis was performed on TGA 4000 System (Perkin Elmer apparatus, Waltham, MA, USA). Briefly, a 20 μL sample was taken for measurement. Each sample was measured in triplicate. The sample was heated from 20 to 150 °C at 10 °C/min in nitrogen atmosphere. The lowest mass was taken as fully dried sample and used for further calculations. The obtained mass was normalized for 20 mg of the initial sample mass. The mean of three measurements was calculated. Density was derived from weighing 5 × 15 μL of the sample and dividing the mean mass by volume. Final concentrations were: SPION-DHP = 3.52 mg/mL and SPION-PEG = 3.81 mg/mL.

### 2.5. HBc Production and Preparation

HBc was produced in plants via a transient expression system based on agroinfiltration. HBc expression vector was constructed on the basis of pEAQ-HT plasmid, developed by Peyret and Lomonossoff [33]. The coding sequence of HBcAg of 552 bp in length derived from HBV subtype *adw4* (GenBank: Z35717), was cloned into the vector *Age* I and *Xho* I restriction sites using sites *Age* I and compatible ends of *Sal* I, respectively, introduced by PCR using the following primers:

Forward: AACCGGTATGGACATTGACCCTTATAAAGAATTTG

Reverse: TGTCGACTGCAGTTAACATTGAGATTCCCGAGATTGAG

Complete vector pEAQ-HBc was introduced into *Agrobacterium tumefaciens* EHA105 and LBA4404 strains via electroporation.

Agroinfection was performed with *Agrobacterium* strains grown overnight on selective liquid LB medium supplemented with kanamycin (50 mg/l) and used to infiltrate leaves of 5–7 week-old *Nicotiana benthamiana* plants, cultivated in growth chamber under 5–6 klx light intensity, 16/8 h photoperiod and at a 22/16 °C temperature regime. *Agrobacterium* cells were centrifuged at 2000 g for 3 min at 4 °C and resuspended in MES buffer (10 mM 2-(N-morpholino)ethanesulphonic acid, 10 mM MgSO_4_, pH 5.7) to optical density at a 600 nm wavelength (OD_600_) 0.6 or 0.1 for infiltration by syringe or exsiccator, respectively. *Agrobacterium* suspension, 0.5 mL per leaf, was injected with a syringe into the bottom side of the leaves. Alternatively, whole plants were inverted and immersed in 2 L of *Agrobacterium* suspension in exsiccator (Lab Companion VDP-25G, Seoul, Korea). Pump (AGA Labor PL2, Poznań, Poland) was then applied to reach underpressure (−0.08 MPa) for approximately 1 min. The vacuum was released and applied again to ensure infiltration of the whole leaves. After 10 days following the agroinfiltration concentration of HBc in plant tissue reached approximately 1 mg/g of fresh weight (data not shown). HBc was then extracted and partially purified using sucrose density gradient as described previously [33]. The concentration of HBc directly after purification was fixed to 0.1 mg/mL. Prior to SPION encapsulation, HBc was diluted twice in a disassembly buffer.

### 2.6. SPION-HBc Preparation

VLPs were prepared in line with a slightly modified procedure described elsewhere [28].

HBc dissociation: 300 μL of 0.1 mg/mL HBc were diluted with 300 μL of denaturant solution (5 M urea, 300 mM NaCl, 100 mM tris-HCl) and incubated at 25 °C for 3h.

SPION-HBc assembly: The solution of dissociated HBc was divided into 100 μL aliquots (HBc conc. 0.05 mg/mL). To each aliquot, functionalized SPIONs were added to a final concentration of 0.5, 1.0 and 2.0 mg/mL. Obtained solutions were dialyzed twice against 400 mL of assembly buffer (150 mM NaCl, 10 mM CaCl_2_, 1% *w*/*v* glycine, 10% *v*/*v* glycerol, 50 mM tris-HCl, pH = 8) for 24 h at 4 °C.

### 2.7. VLPs Antigenicity

Antigenicity of HBc VLPs was assessed via enzyme-linked immunosorbent assay (ELISA). HBc assembled with functionalized SPION-PEG and SPION-DHP at different concentrations (mg/mL) in comparison to the standard protein (recombined in *E. coli*, Cat No. R8A120, Meridian Life Science Inc., Memphis, TN, USA). Antigenicity defined as absorbance at 405 nm of two-fold dilution series of VLPs (from 1:160 to 1:81,920) and standard protein (from 0.5 to 0.004 µg/mL).

### 2.8. Statistical Analysis

Results of SPION-HBc formation were analyzed using a two-way ANOVA followed by a Duncan test; differences were considered significant at *p* ≤ 0.05. Statistical analysis was performed using the Statistica 8.0 statistical software package (StatSoft Inc., Tulsa, OK, USA).

### 2.9. Characterization Methods

Transmission electron microscopy (TEM) images were acquired with Hitachi TEM HT7700 microscope (Tokyo, Japan). Grids were made of copper coated with a carbon film, mesh 300. Samples were prepared by placing 15 μL drop on the grid and draining the excess solution with blotting paper and left for 15 min. to dry. Subsequently, samples were negatively stained with 10 μL of 2% uranyl acetate. Particle size analysis was performed with free ImageJ software version 1.51w (NIH, Bethesda, MD, USA).

Dynamic light scattering (DLS) and ζ-potential measurements were performed on Malvern Zetasizer Nano ZS90 (Worcestershire, UK) in a Folded Capillary Zeta Cell DTS1070. Prior to measurement, samples were briefly sonicated, diluted to optimal concentration and filtered with a 0.2 μm syringe filter (Merck Millipore, Burlington, MA, USA). Measurements were repeated in triplicate.

ELISA was performed on the assembled HBc VLPs, with or without SPION core. The assay was performed in line with a procedure described previously [34]. MaxiSorp (NUNC) 96-well microplate was coated overnight at 4 °C with of HBc-specific mAb (0.5 mg/mL) (Cat. No. C31190 M, Meridian Life Science Inc., Memphis, TN, USA) in carbonate buffer pH 9.6. Each step following the coating was preceded by three washes with PBST buffer (phosphate buffered saline with additional 0.05% *v*/*v* Tween20, Sigma, Saint Louis, MO, USA). The coated wells were blocked for 1 h with 5% (*w*/*v*) fat-free milk/PBS, followed by incubation with 100 µL of antibody solution for 1 h at 25 °C. The samples were added to the PBS-filled wells and two-fold serially diluted. HBc produced in *E. coli* (Cat. No. R8A120, Meridian Life Science) was used as the reference. Rabbit polyclonal PBST antibody specific to HBc (Cat. No. LS-C67451/18649, Life Span Biosciences, Seattle, VA, USA) 0.125 mg/mL and goat anti-rabbit whole-molecule polyclonal antibody AP-conjugated (Sigma) 1:10,000 dilutions were premixed and added as the primary and secondary antibody. Finally, the substrate for alkaline phosphatase (pNPP, Sigma) was added and the reaction was developed at 25 °C for at least 30 min. The absorbance was measured at 405 nm using a microplate reader (Model 680, Bio-Rad, Hercules, CA, USA).

## 3. Results

### 3.1. SPIONs Synthesis and Functionalization

**Figure 1:**
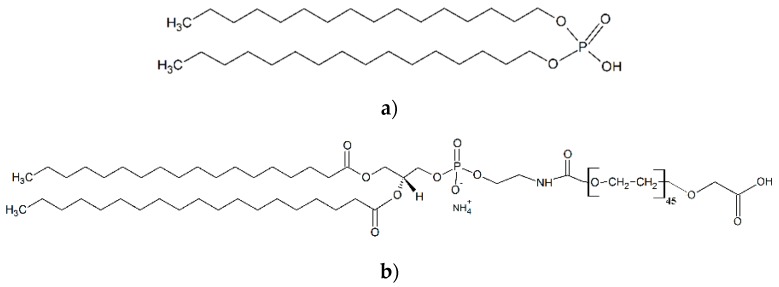
Molecular structure of ligands used for superparamagnetic iron oxide nanoparticles (SPIONs) functionalization. (**a**) dihexadecyl phosphate (DHP); (**b**) 1,2-distearoyl-sn-glycero-3-phosphoethanolamine-N-[carboxy-(polyethylene glycol)-2000] (PL-PEG-COOH).

Monodispersed superparamagnetic iron oxide nanoparticles (SPIONs) of 15 nm diameter were synthesized via thermal decomposition of iron (III) acetylacetonate Fe(acac)_3_ (Figure S1). The approximate diameter of the HBc VLP internal cavity is 25 nm for the T = 4 particles and 21 nm for the T = 3 [14]. Therefore, both structures provide sufficient marginal space to accommodate the ligands. In order to obtain negative surface charge, SPIONs were functionalized with short and long chain ligands: dihexadecyl phosphate (DHP) and 1,2-distearoyl-sn-glycero-3-phosphoethanolamine-N-[carboxy-(polyethylene glycol)-2000] (PL-PEG-COOH) (Figure 1a,b). Functionalized SPIONs will be denoted as SPION-DHP and SPION-PEG, respectively.

DHP functionalization was performed according to our previously described method [31]. PL-PEG-COOH functionalization was achieved by a slightly modified protocol by Huan et al. [24]. Both SPION-DHP and SPION-PEG were analyzed via ζ-potential and dynamic light scattering (DLS) measurements (Table 1). In both cases, functionalization is driven by hydrophobic interactions between oleic acid residues present on the surface of as-obtained SPIONs and alkyl chains of the ligands. More detailed characterization of both functionalizations can be found in our previous work [31].

Despite rather small differences in surface charge, hydrodynamic radius differs substantially. Counterintuitively, long-chain PEG ligand provided smaller hydrodynamic radius than DHP, which may be partially caused by differences in ζ-potential. Another plausible explanation is the interplay of surface charge and multilayered micelle structure formation.

### 3.2. VLPs-SPION Assembly

**Figure 2:**
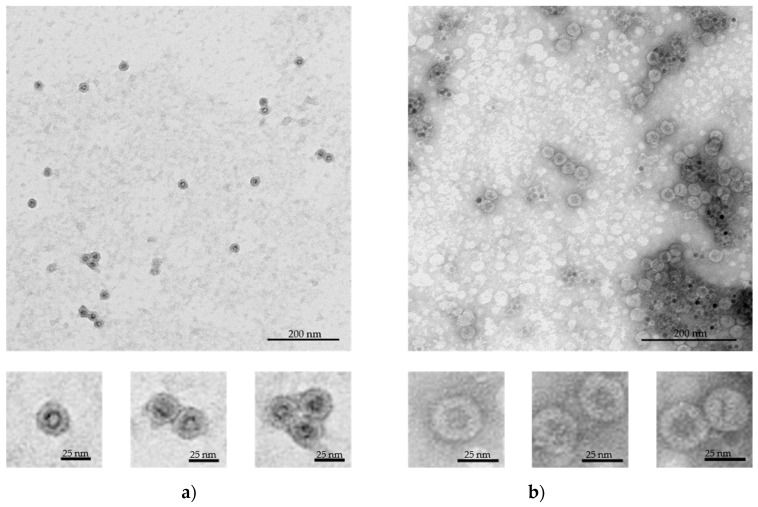
Transmission electron microscopy (TEM) images of the assembled VLPs with magnetic cores, negatively stained with 2% uranyl acetate. (**a**) SPION-DHP-HBc VLPs obtained at 0.2 mg/mL of SPION-DHP and 0.05 mg/mL of HBc; (**b**) SPION-PEG-HBc VLPs obtained at 0.05 mg/mL of SPION-PEG and 0.05 mg/mL of HBcAg.

The assembly rates and core encapsidation efficiency are strictly dependent on surface charge density, capsid protein concentration and core/capsid protein stoichiometric ratio [35]. Therefore, HBc concentration was fixed at 0.05 mg/mL while SPIONs concentrations were varied between: 0.05, 0.1 and 0.2 mg/mL. It is important to note that due to differences in ligands’ molecular weight and probable differences in functionalization densities, equal *w*/*v* concentrations of SPION-PEG and SPION-DHP do not represent the same amount of particles in the solution. The most successful core-VLP assembly was obtained at following concentrations: 0.2 mg/mL SPION-DHP and 0.05 mg/mL SPION-PEG (Figure 2a,b).

TEM images were used to measure the diameter of the assembled VLPs. Mean diameter of SPION-DHP-HBc was 28.4 ± 1.2 nm while SPION-PEG-HBc mean diameter was 29.9 ± 1.5 nm (Table 2). The obtained measurements indicate that in both cases capsids assembled into T = 3 symmetry (native size of T = 3 capsid is 30 nm). Nonetheless, to assess the VLPs symmetry with certainty, crystallographic studies would be required. The obtained results are concordant with thermodynamic studies of nanospheres encapsulated in virus capsids reveling, in that core surface charge and its radius determine the size of the capsid formed around the nanoparticle [36]. In this case, despite T = 4 symmetry being a predominant form of in vitro HBc self-assembly (~95%), introduction of SPION-DHP and SPION-PEG facilitated assembly into a smaller, presumably T = 3 form. This may suggest that negative surface charge density was high enough to drive the assembly into less energetically-favorable capsid morphology. Studying TEM images, SPION-DHP assembly displayed higher efficiency than SPION-PEG and resulted in minority of empty capsids and unassembled cores. In comparison, SPION-PEG assembly produced a multitude of empty capsids along with unassembled cores.

### 3.3. VLPs ELISA

**Figure 3:**
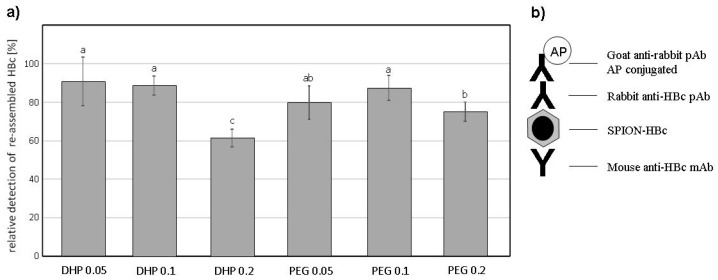
(**a**) HBc re-assembly on SPIONs functionalized with DHP or PEG in different concentrations (mg/mL) in comparison to the initial preparation of plant-derived antigen (100%). Statistically significant differences marked by a letter indexes; (**b**) Scheme of enzyme-linked immunosorbent assay (ELISA) test used for assay of VLP-assembled HBc and SPION-HBc VLPs. AP—alkaline phosphatase.

Antigenicity of the obtained VLPs was assessed via ELISA (Figure 3 and Figure S4). Despite unvaried HBc protein concentrations, for all VLPs variants decreased signals of HBc detection in comparison to control (initial HBc used) were observed, as well as some significant differences in signals among VLPs variants were found. All concentrations of SPION-PEG displayed a decrease in signal intensity; however, the differences between 0.05 and 0.1 mg/mL concentrations were not statistically significant. Additionally, in comparison to analogous variants of SPION-DHP. For SPION-PEG, the highest observed amount of core-VLPs was found at 0.05 mg/mL (Figure 2B), which also resulted in decreased signal intensity, although insignificantly different from other SPION-PEG concentrations. Finally, among all variants of SPION-HBc VLPs, the significantly lowest signal, 61.6% of HBc was recorded for 0.2 mg/mL SPION-DHP, the same concentration at which the highest number of core-VLPs was observed. The obtained results indicate that core introduction into HBc derived VLPs may decrease antigenicity. This phenomenon could be explained by the proclivity of HBcAg to assemble into smaller T = 3 capsids in the presence of the SPIONs, which in turn results in higher antigen density on the VLPs surface and competitive binding of antibodies. A study by Wu et al. has shown that the amount of antibodies bound to the capsid depends on its morphology and is significantly decreased for T = 3 capsids [37]. Moreover, steric hindrance has been proven to be a crucial factor for antibody binding to surface antigens [38,39]. These results provide a great starting point for further investigations of the relationship between core properties, capsid morphology, antigenicity and biological activity of the HBc derived VLPs.

## 4. Discussion

Core-VLPs assembly, here SPION-HBc, is a kinetically complex cascade of interactions between viral proteins, nanoparticle surface and an ionic environment. In silico modeling predicts that core introduction provides a plethora of advantages such as increased assembly rates and efficiency over wider set of conditions, stimulation of the assembly below critical subunit concentration (CSC), possibility of templating VLPs morphology. Nonetheless, computational modeling results are not always confirmed in experimental studies. For example, the predicted increase of assembly efficiency driven by the increase of surface charge density has been overestimated in comparison to experimental data [35]. Moreover, simulations predominantly assume cores geometry perfectly commensurate with capsid interior. In our case, functionalized 15 nm SPIONs were not perfectly fitted into the capsid, which could elicit the existence of kinetic traps, not predicted by the computational studies [40]. The most successful core assembly was achieved at a 0.2/0.05 mg/mL core/protein ratio with SPION-DHP. Slightly lower ζ-potential concomitant with larger hydrodynamic diameter in comparison to SPION-PEG might indicate that surface charge density was higher in case of SPION-DHP. SPION-PEG assembly at 0.05/0.05 mg/mL core/protein ratio resulted in partially successful core assembly along with multitude of empty capsids and unassembled cores. This result indicates that one or several assembly parameters were suboptimal; however, due to the complexity of the process, we are unable to pinpoint the exact cause of lower efficiency. It is possible that interactions between less negatively charged SPION-PEG and positively charged domains of the HBc were insufficient to win competition over subunit-subunit attraction, resulting in the empty capsids. Additionally, it is important to note that the ligands used differed in length which could also affect assembly kinetics. In both cases, core introduction resulted in slightly smaller VLPs diameters even for T = 3 capsid morphology, which could be attributed to more compact HBc dimer-dimer spacing resulted from strong electrostatic core-HBc dimer interactions. This thesis stands in agreement with our experimental data showing smaller core-VLPs diameters for lower values of the core’s ζ-potential (Table 2). VLPs morphology driven antigenicity is a crucial aspect determining its potential application, especially in the area of vaccinology. In that respect, our study demonstrated successful assembly together with substantially retained HBc antigenicity, although decreased in comparison to native HBc protein preparation containing mainly T = 4 capsids (Figure 3). This may indicate competitive binding of antibodies and/or steric clashes due to increased antigen surface density, stemming from the decreased VLPs diameter. Crucially, for many medical applications such as cell- or tissue-specific targeting, decreased immunogenicity of core antigen may be desirable. Nonetheless, this theory requires more in depth experimental investigation to be confirmed. Surface charge density is one of the most important parameters guiding core assembly. However, it is not easily measureable by standard lab equipment, which impedes rational design of physicochemical properties of the core. Therefore, we propose the use of ζ-potential and hydrodynamic diameter, as two parameters encompassing surface charge density. This approach would simplify and unify core’s surface electrostatic characterization, providing more accessible tool for core-VLPs design.

## 5. Conclusions

This study provides an experimental assessment of the crucial parameters guiding SPION-HBc VLPs assembly and evaluates antigenicity of the obtained structures. The presented results highlight potential directions for further studies regarding the mechanism guiding HBc VLPs assembly with metallic cores as well as their antigenic properties.

## Figures and Tables

**Table 1:** ζ-potential and hydrodynamic radius of the functionalized superparamagnetic iron oxide nanoparticles (SPIONs) obtained by dynamic light scattering (DLS) (Figures S2 and S3).

	SPION-DHP	SPION-PEG
ζ-potential	−44.0 ± 3.4 mV	−37.3 ± 2.9 mV
Hydrodynamic diameter	53.75 ± 1.93 nm	29.69 ± 1.57 nm

**Table 2:** VLPs diameter measurements obtained from transmission electron microscopy (TEM) images. Measured with ImageJ software.

	SPION-DHP-HBc	SPION-PEG-HBc
Diameter	28.4 ± 1.2 nm	29.9 ± 1.5 nm
Number of measured VLPs	41	19

## Supplementary Materials

The following are available online at http://www.mdpi.com/2079-4991/9/2/155/s1, Figure S1: TEM images of as synthesized SPIONs, Figure S2: DLS results for SPION-PEG and SPION-DHP, Figure S3: ζ-potential for SPION-DHP and SPION-PEG, Figure S4: ELISA of HBcAg SPION assembly.
