# Supplementary material for: Assembly and Characterization of HBc Derived Virus-like Particles with Magnetic Core
Source: Nanomaterials (Basel). 2019 Jan 26;9(2):155. doi: 10.3390/nano9020155 (PMC6409934; doi:10.3390/nano9020155)
Supplement: Supplementary file 1 [file nanomaterials-09-00155-s001.pdf]

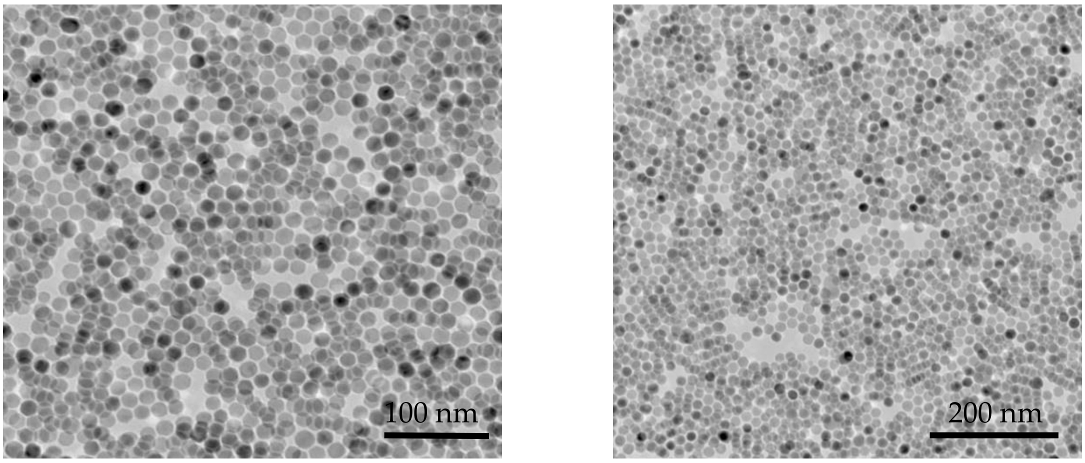

Figure S1. Transmission electron microscopy (TEM) image of 15 nm SPIONs synthesized via thermal decomposition of iron (III) acetylacetonate Fe(acac)3.

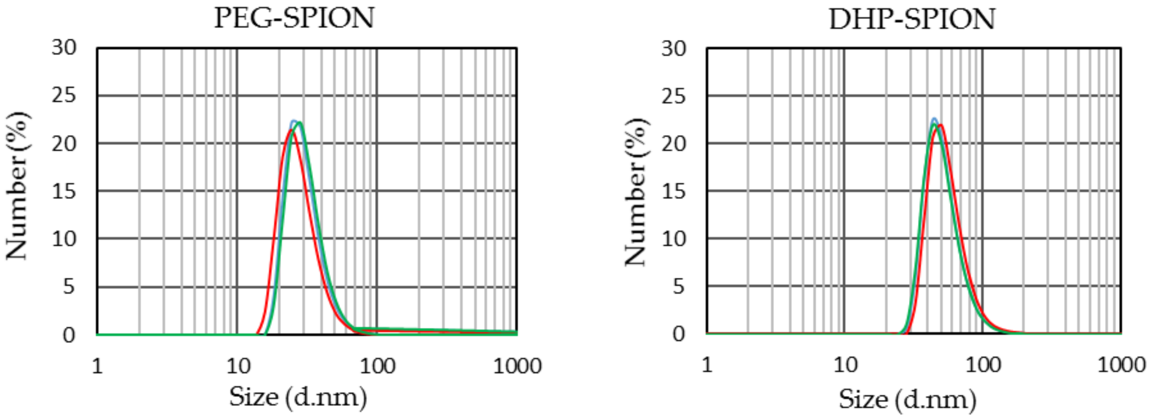

Figure S2. Dynamic light scattering measurement (DLS) of hydrodynamic diameter. SPION-PEG: Number mean: 29.69 ± 1,57 nm; Polydispersity index (PdI): 0.16. SPION-DHP: Number mean: 53.75 ± 1.93 nm; Polydispersity index (PdI): 0.154. Measurements performed in triplicate.

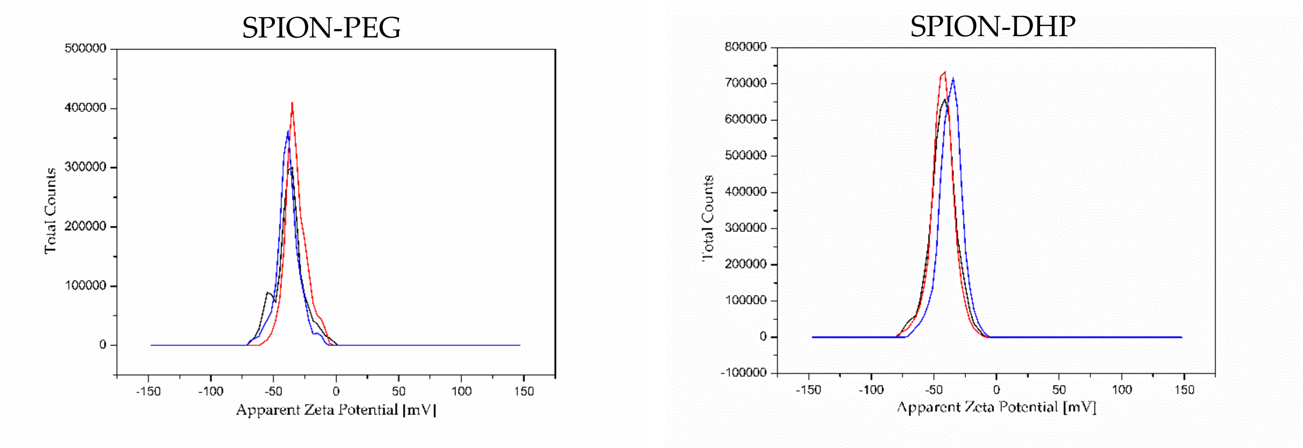

Figure S3. ζ-potential measurements. SPION-PEG: -37.3 ± 2.9 mV; SPION-DHP: -44.0 ± 3.4 mV.

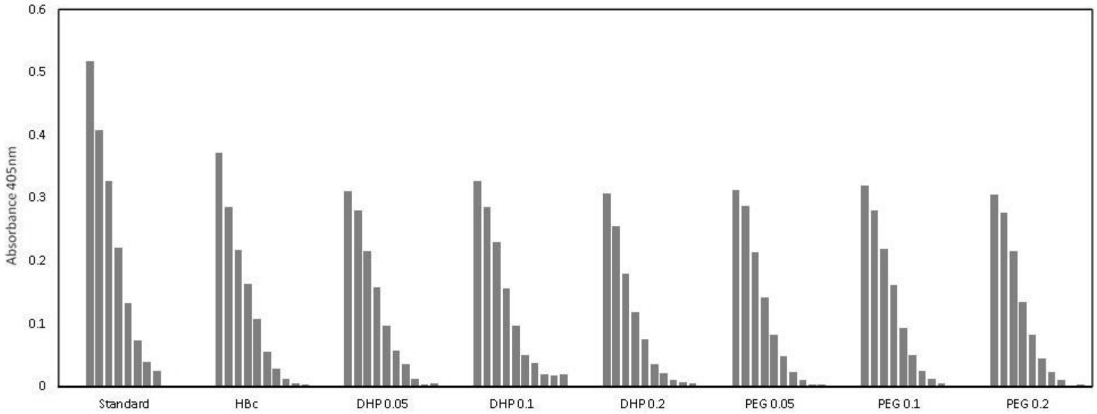

Figure S4. Antigenicity of SPION-HBc VLPs obtained at different SPION/HBc concentrations in comparison to standard protein, assessed by ELISA.
